# Full Digital Workflow in the Esthetic Dental Restoration

**DOI:** 10.1155/2022/8836068

**Published:** 2022-06-18

**Authors:** Suchada Kongkiatkamon, Dinesh Rokaya

**Affiliations:** ^1^Bangkok Hospital Dental Center, Bangkok Hospital, Bangkok 10310, Thailand; ^2^Department of Clinical Dentistry, Walailak University International College of Dentistry, Walailak University, Bangkok 10400, Thailand

## Abstract

This case report presented a fully digital workflow in esthetic dental restoration. A 51-year-old female patient was referred to BDMS Wellness Clinic due to a fracture of the maxillary left central incisor. An immediate dental implant was planned to restore tooth 21 with esthetic crown lengthening of upper front teeth and new zirconia crowns for teeth 11, 12, 13, 21, 22, and 23. Digital impression was made using a digital scanner (PRIMESCAN®, Dentsply Sirona, Bensheim, Germany); surgical guide (Cerec Guide 3, Dentsply Sirona, Bensheim, Germany) was designed by using a designing software (Galileos Software, Dentsply Sirona, Bensheim, Germany) and was milled by using a milling machine (MCXL milling machine, Dentsply Sirona, Bensheim, Germany) to create a precise surgical guide. 3D smile design was done by using the Digital Smile Design (DSD) program, the crown lengthening guide was designed according to DSD designed by using a designing software (Inlab 19 Software, Dentsply Sirona, Bensheim, Germany), and the guide was fabricated by a 3D printer (FormLabs Form 2, Formlab, MA, USA). Provisional crowns (splinted) for teeth 11, 12, 13, 21, 22, and 23 were milled by using polymer (VIPI BLOCK TRILUX®, VIPI Industria, Pirassununga, SP, Brazil) using a milling machine (MCX5, Dentsply Sirona, Bensheim, Germany). The zirconia crowns were designed by using software (Inlab19 Software, Dentsply Sirona, Bensheim, Germany) and milled using the same milling machine. At the implant position, Ti-base is cemented by using an abutment (Multilink Hybrid Abutment, Ivoclar Vivadent, Schaan, Lichtenstein, Germany) with zirconia coping (Cercon base white shade, Dentsply Sirona, Bensheim, Germany) utilized as the abutment. The zirconia crowns (Cercon Xt, Dentsply Sirona, Bensheim, Germany) were sintered and characterized and then cemented. The patient was satisfied with the esthetic outcome of the treatment.

## 1. Introduction

Clinical success in dentistry is highly influenced by the materials, techniques, and proper planning [1]. Recently, digital dentistry has been increasingly used in restorative and prosthetic dentistry due to advancements in technologies, like intraoral scanners (IOS) and software [2, 3]. Using such technologies has also allowed the dentists to work more efficiently and precisely reducing working time using in-house computerized techniques [1, 4]. In addition, incorporating digital technologies in clinical dentistry has enhanced success [5].

The Digital Smile Design (DSD) is a recently invented digital tool that provides esthetic rehabilitation planning improving the outcome of the treatments [6]. The DSD can be performed in 2D or 3D digital processes in an entire digital flow. The video documentation simplifies and facilitates the documentation procedure, facial analysis, smile design, treatment planning, communication, and patient education [7]. The DSD can be done in both conventional or virtual models with the subsequent fabrication of the prostheses, i.e., computer-aided design and computer-aided machine (CAD-CAM) restorations [8–11].

There is always a challenge to improve esthetics while maintaining the high strength of the restorative materials. Still, zirconia has been attractive in restorative and prosthetic dentistry due to its esthetic and strength [12, 13]. There is progressive development of currently available and next-generation zirconias towards greater translucency while preserving adequate strength and toughness. In past, glass ceramics were popular restorative materials because of their translucencies similar to natural teeth, but they did lack the adequate strength requirements [14, 15]. Later, the zirconia improved esthetically, where restorations were made from single materials with no layering ceramic. These varieties of zirconia were strong, but the esthetics were still not ideal. At present, zirconia has the esthetic properties desirable for the ideal restorative material, in addition to being strong. The favorable properties of zirconia for dental implantology include biocompatibility, osseointegration, favorable soft-tissue response, and esthetics [16, 17].

The current zirconia is yttrium-stabilized tetragonal zirconia polycrystals (Y-TZP), zirconia-toughened alumina (ZTA), and alumina-toughened zirconia [18–20]. Y-TZP is predominantly used for the fabrication of dental crowns and fixed prostheses. The Y-TZP's grain size affects the final product's stability and mechanical properties. If the grain size is too large, it is less stable, and if the grain size is too small, it may result in reduced fracture toughness [21, 22]. Y-TZP can vary depending on additives and dopants, sintering profiles, and ensuing heat treatments [23, 24]. This case report presents a fully digital workflow, and a new generation of zirconia was used in the esthetic dental restoration.

## 2. Case Report

**Figure 1:**
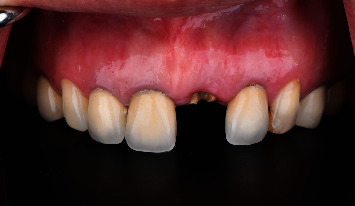
Pretreatment clinical photograph showing the fracture of tooth number 21 almost below the cementoenamel junction with no ferrule effect.

**Figure 2:**
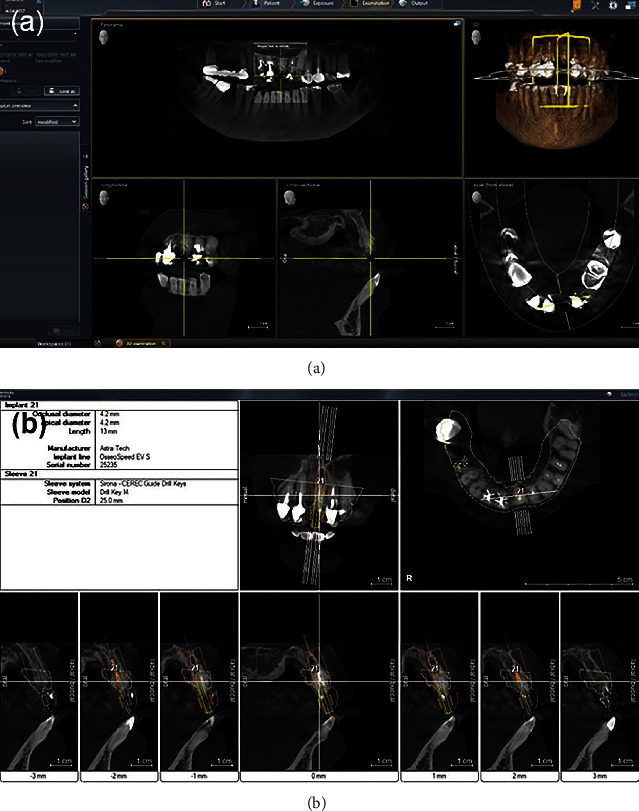
Radiograph for the diagnosis and treatment planning (a, b).

A 51-year-old female patient was referred to our clinic due to a fracture of the maxillary left central incisor. Regarding her past dental history, she had several anterior restorations done over 15 years ago. Her incisor was fractured due to accidentally biting on a peanut. On intraoral examination, tooth number 21 was fractured almost below the cementoenamel junction (CEJ) with no ferrule effect (Figure 1), and secondary caries was found around the margins of teeth 11, 12, 13, 21, 22, and 23. On radiographs, tooth 21 also showed no periapical radiolucency (Figure 2(a)).

As the tooth could not be restored, three treatment plan details were proposed to the patient for the fractured central incisor after its extraction; (i) 3-unit bridge, (ii) removable partial denture, and (iii) single dental implant-supported crown. The patient chose the dental implant option. In addition, she also mentioned that she wanted to correct her gummy smile. Hence, the treatment sequences were planned as the periodontal treatment, followed by esthetic crown lengthening of upper front teeth and immediate implant placement. In addition, new zirconia crowns were planned for teeth 11, 12, 13, 21, 22, and 23. A computed tomography (CT) scan was taken using Orthophos SL 3D (Dentsply Sirona, Bensheim, Germany) (Figure 2(b)). The patient was informed about all procedures with risks and benefits, and consent was taken for all the procedures.

**Figure 3:**
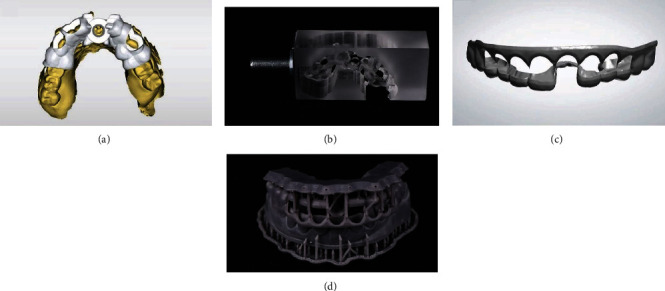
Design and fabrication of guides. Design of implant surgical guide in software (a), milled implant surgical guide (b), design of esthetic crown lengthening guide (c), and 3D printed esthetic crown lengthening guide (d).

Digital impression was made using a digital scanner (PRIMESCAN Dentsply, Sirona, Bensheim, Germany). Then, a surgical guide (Cerec Guide 3, Dentsply Sirona, Bensheim, Germany) was designed by using a designing software (Galileos Software, Dentsply Sirona, Bensheim, Germany) and was milled using a milling machine (MCXL milling machine, Dentsply Sirona, Bensheim, Germany) to create a precise surgical guide (Figure 3). 3D smile design was done by using Digital Smile Design (DSD), crown lengthening guide was designed according to DSD design by using a designing software (Inlab 19 software, Dentsply Sirona, Bensheim, Germany), and the guide was fabricated by a 3D printer (FormLabs Form 2, Formlab, MA, USA).

**Figure 4:**
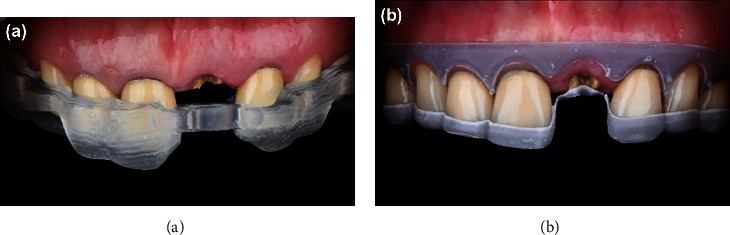
Try-in of implant surgical guide (a) and crown lengthening guide (b).

On the surgical day, the dental implant surgical guide and the crown lengthening guide were tried in, and the fit and accuracy were verified (Figure 4). Then, a local anesthetic agent was given adequately, and crown lengthening was performed according to the crown lengthening guide.

**Figure 5:**
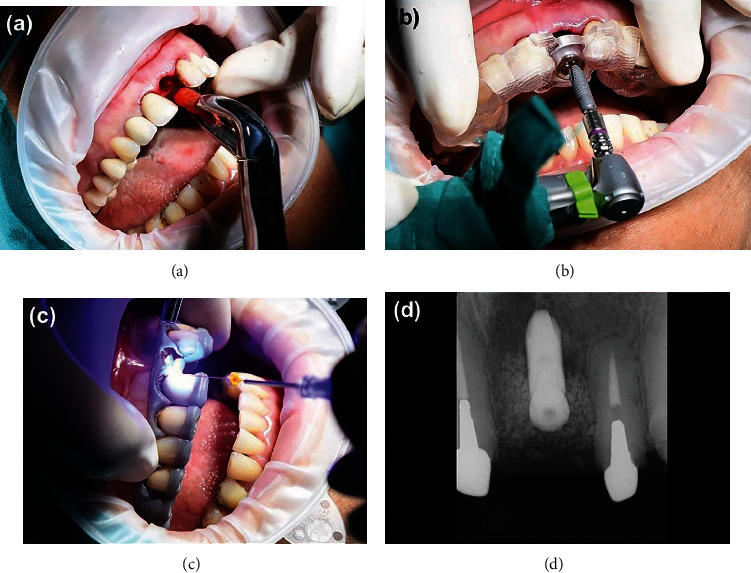
Atraumatic extraction of tooth 21 (a), dental implant placement using a surgical guide (b), crown lengthening by using a laser (SiroLaser Blue Dentsply Sirona, Bensheim, Germany) with digitally assisted crown lengthening guide (c), and periapical radiograph post dental implant placement (d).

Atraumatic extraction of tooth 21 was done, and an immediate dental implant (Astra tech OsseoSpeed™ EV, Dentsply Sirona, Bensheim, Germany) of size 4.2 × 11 mm was inserted using the surgical guide, and a periapical radiograph was taken following dental implant placement (Figure 5).

3D smile design was done by using Digital Smile Design (DSD) (DSDApp LLC). DSD is a very unique way to help both clinicians and patients to have an idea of what will happen in the future of their smiles, and the software was created by Dr. Coachman [7, 25]. The clinicians need to take the patient face and smile photograph; also, digital teeth impression and all documents will be merged and planned together in the DSD software [7].

Then, crown lengthening used a laser (SiroLaser Blue Dentsply Sirona, Bensheim, Germany) with a digitally assisted crown lengthening guide. The primary stability was checked, and it was adequate, and a periapical radiograph was taken to check the position. Spongious bone substitute small granules (0.25 mg) (BioOss, Geistlich Biomaterials, Sweden, Switzerland) were also utilized to augment the labial jumping gap. The cover screw was placed on the implant.

**Figure 6:**
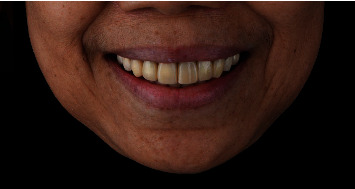
Provisional crowns in the patient's mouth on the day of the surgery.

The porcelain fused to metal crowns on teeth 11, 12, 13, 21, 22, and 23 was sectioned and removed. Caries was removed, and teeth preparation was performed. Digital impression was made with PRIMESCAN® (Dentsply Sirona, Bensheim, Germany). Provisional crowns (splinted) for teeth 11, 12, 13, 21, 22, and 23 were made by milling (VIPI BLOCK TRILUX®, VIPI Industria, Pirassununga, SP, Brazil) using the milling machine (MCX5, Dentsply Sirona, Bensheim, Germany) (Figure 6). For tooth number 21 (implant site), the pontic shape was designed to mold the soft tissue contour. The provisional crowns were inserted and recalled after 1 week for a recheck.

**Figure 7:**
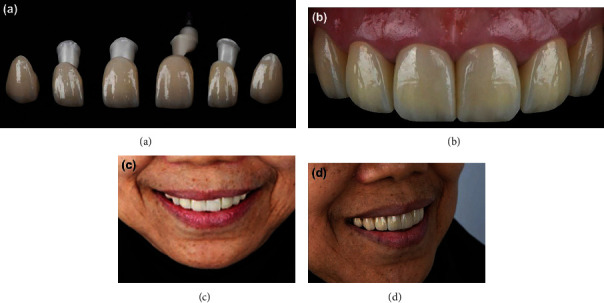
Final restorations. Zircona crowns (a), cemented in the patient (b), front view of the crowns in the patient (c), and side view of the crowns in the patient (d).

Following 4 months of dental implant placement, the patient was called to the clinic for the final restoration. Final tooth preparation was done for teeth 11, 12, 13, 21, 22, and 23. Scan Post was used to digitally capture the position of the implant intraorally. Then, the digital impressions were made using a digital scanner (PRIMESCAN®, Dentsply Sirona, Bensheim, Germany) and sent to the dental laboratory for the fabrication of the final crowns. Zirconia block with A2 shade (Cercon Xt, Dentsply Sirona, Bensheim, Germany) was selected and used for crowns. The zirconia crowns were designed by using software (Inlab19 Software, Dentsply Sirona, Bensheim, Germany) and milled by using MCX5. At the implant position, Ti-base is cemented by using an abutment (Multilink Hybrid Abutment, Ivoclar Vivadent, Schaan, Lichtenstein, Germany) with zirconia coping (Cercon base shade White, Dentsply Sirona, Bensheim, Germany) utilized as the abutment. Zirconia crowns were sintered and characterized (Figure 7(a)). The completed zirconia crowns were cemented in the patient with resin cement (Rely X Ultimate, 3M ESPE, Dental Products, St. Paul, MN, USA) following the manufacturer's instructions (Figure 7(b)). The patient was satisfied with the outcome, and the patient has been recalled for a recheck (Figures 7(c) and 7(d)). At the 10-month follow-up, the patient was satisfied and did not have any complaints.

## 3. Discussion

Digital technology is transforming every aspect of our world. It makes the patient's visit more efficient and more pleasant. At present, with 3D modeling and software such as CAD-CAM, the dentist can plan restorative procedures easily with fewer errors [26]. IOS, apart from obtaining impressions, detects the obtained impressions suitable for the modeling of restorations [27]. Digital impressions contribute to the precise registration of the bite and help to skip various procedures that may cause distortions [28].

In our case, every procedure (from impression to final restoration) was planned digitally by using facial analysis and dynamic smiles and integrated all information. The advantage of digital planning is to be able to generate the preview picture and smile prototype for the patient, so the patient can see the outcome before the operation starts. Other than that, the full digital workflow also helps us to eliminate irrelevant procedures that could potentially create errors in our treatment too. The huge benefit of the digital workflow is to help us to learn from our case and also help us to be able to repeat or reproduce our work easily in case any of the restorations need to be replaced in the future.

In this case report, we used newer zirconia (Cercon Xt), and it demonstrated extra high translucency with life-like esthetics and good flexural strength (750 MPa) [29]. This zirconia has the composition: zirconium oxide, yttrium oxide (9%), hafnium oxide (<3%), aluminium oxide, and silicon oxide (<1%). The translucency of monolithic Y-TZP ceramics plays an important role in the esthetic of anterior tooth restoration. The factors affecting the translucency of monolithic Y-TZP ceramics include restoration thickness, the color of the monolithic zirconia, surface finishing methods, low-temperature degradation, wear, cementation type, dental background, and cement color [19].

Currently, the greatest challenge is to produce them with sufficient esthetics to match existing dentition. In this regard, Y-TZP has to compete with more translucent but weaker glass ceramics, notably the lithium-based silicates [30]. Regarding the use of such new zirconia materials, more clinical studies with follow-up of the final zirconia restorations are necessary for the long-term results of the digital workflow. For our case, a long-term follow-up is needed, even if the patient is satisfied with the esthetic outcome.

## 4. Conclusion

This case report presents a fully digital workflow in esthetic dental restoration. The author used digital technology as one of many communication tools to make sure all procedures/steps went according to the plan. Errors from conventional methods were eliminated. The number of visits and amount of time decreased by combining various procedures. In esthetic rehabilitation, proper treatment plans and surgical and prosthetic procedures guided by the diagnostic procedures help in the clinical success of the final restoration. In this case, the patient was fully satisfied with the esthetic and function.

## Data Availability

The data used to support the findings of this study are available from the corresponding author upon reasonable request.
